# How I do it: continuous intraventricular interferon alpha infusion in pediatric patients with subacute sclerosing panencephalitis

**DOI:** 10.1007/s00701-025-06693-3

**Published:** 2025-11-08

**Authors:** Francesco Tengattini, Gabriella Errichiello, Antonio Varone, Giuseppe Cinalli, Claudio Ruggiero

**Affiliations:** 1 Neurosurgery, Department of Medical and Surgical Specialties, Radiological Sciences and Public Health, University of Brescia and Spedali Civili Hospital, Brescia, Italy; 2 Neurosurgery, Department of Neurosciences, Santobono-Pausilipon Children’s Hospital, AORN, Naples, Italy; 3 Pediatric Neurology, Department of Neurosciences, Santobono-Pausilipon Children’s Hospital, AORN, Naples, Italy; 4 Child Neuropsychiatry Unit, Department of Translational Medical Science, University of Naples Federico II, Naples, Italy

**Keywords:** Subacute sclerosing panencephalitis, Interferon alpha therapy, Intraventricular infusion, Rechargeable pump, Step-by-step surgery, 2-Dimensional operative video

## Abstract

**Background:**

Subacute sclerosing panencephalitis (SSPE) is a chronic disease affecting the central nervous system (CNS) because of persistent measles virus (MeV) infection. Among the various treatment available the intraventricular interferon alpha administration demonstrated greater effectiveness.

**Method:**

In this article is described the step-by-step surgical technique of the positioning of an intraventricular catheter connected to a rechargeable subcutaneous pump. The main surgical steps and the pump settings are illustrated in a supplementary video.

**Conclusion:**

This surgical management guarantees a continuous drug release improving the therapeutic effect in terms of clinical and neuroradiological outcome and reducing the toxicity profile.

**Supplementary information:**

The online version contains supplementary material available at 10.1007/s00701-025-06693-3.

## Introduction and Relevant surgical anatomy

Subacute sclerosing panencephalitis (SSPE) is a rare neurodegenerative condition with progressive and often fatal motor and cognitive decline [3]. It is caused by persistent measles virus (MeV) infection, but its pathogenesis is not fully known [7]. The symptomatology is characterized by a spectrum of symptoms going from psychomotor regression to the onset of seizures, up to akinetic mutism, vegetative state and exitus [5]. Diagnosis is based on Dykens’criteria and typically requires two major and one minor criteria [2]. Brain MRI is often not definitive for diagnosis but should be considered for tracking disease progression [1] and for studying the anatomy in order to plan the optimal surgical treatment. There is currently no unequivocal treatment for SSPE. The drugs used include Isoprinosine monotherapy with a possible combination with ribavirin, intravenous immunoglobulin therapy, amantadine and especially intrathecal α-interferon (α-IFN) [8]. Among different modalities of α-IFN delivery, continuous intrathecal infusion device with a reservoir placed in a subcutaneous lumbar pocket and intraventricular administration via an Ommaya reservoir have been considered and demonstrated great effectiveness [4, 9]. In this study we present the positioning of a rechargeable lumbar pump for continuous intraventricular α-IFN infusion that has rarely been considered in the literature. In order to perform this procedure the ventricular size with different anatomical variabilities must be considered. In addition, the abdominal and cranial sites must be checked to select patients that can underwent the surgical procedure.

## Description of the technique

### Clinical presentation

The management and the steps of the surgery are described using the illustrative case of a 5-year-old male presented in the ER for gait impairment disturbance, left hemiparesis and dysarthria and developing, after 7–10 days, visual disturbance with difficulty in recognizing objects.

Serial MRI scans revealed progressive worsening hyperintensity in T2 FLAIR sequences of the periventricular white matter, corona radiata, semioval centers, basal ganglia, thalami, mesencephalic regions and occipital lobes with a reduction of the trophism of the midbrain, pons, upper and middle cerebellar peduncles. The EEG revealed a deconstruction of electrical background activity, with subcontinuous epileptiform anomalies prevailing over the left frontal and temporal regions. The anamnestic investigation revealed a previous measles infection and elevated measles immunoglobulin peak was detected in both CSF and serum.

A diagnosis of SSPE was then established based on Dykens’ criteria and the patient underwent surgical positioning of a rechargeable pump for continuous intraventricular interferon alpha infusion.

### Surgical steps (VIDEO)

The procedure is performed under general anesthesia and orotracheal intubation, the patient is placed in a supine position with the belly and the ear at the same height to facilitate the tunnelling of the catheter. The surgical site from the head to the homolateral portion of the belly is prepared under aseptic precautions and draped; the navel must be included in the surgical field as a reference.

**Fig. 1:**
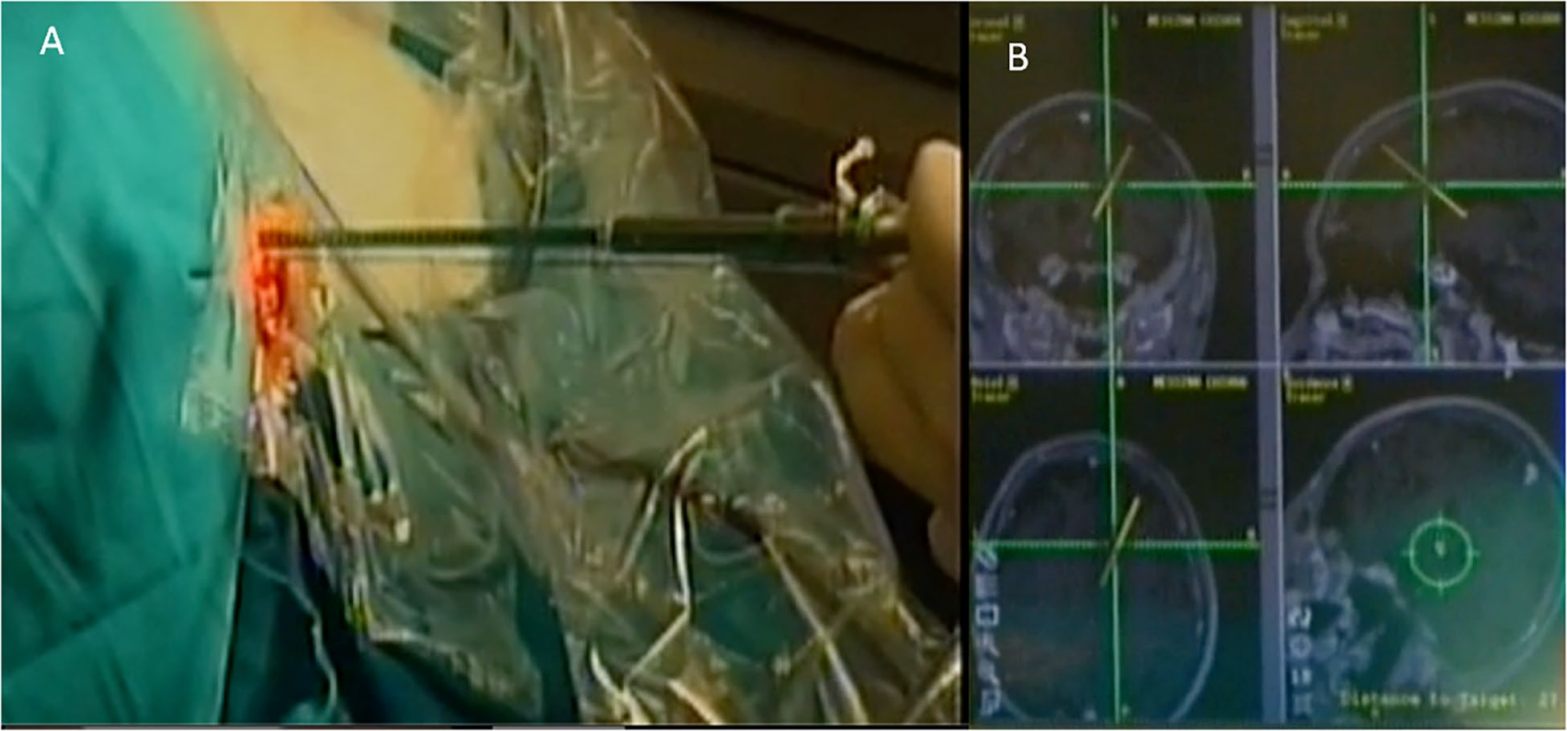
**A** Insertion of the endoscope into the burr hole with the help of the neuronavigation stylet. **B** The neuronavigation monitor is used to follow the planned trajectory to the right lateral ventricle

**Fig. 2:**
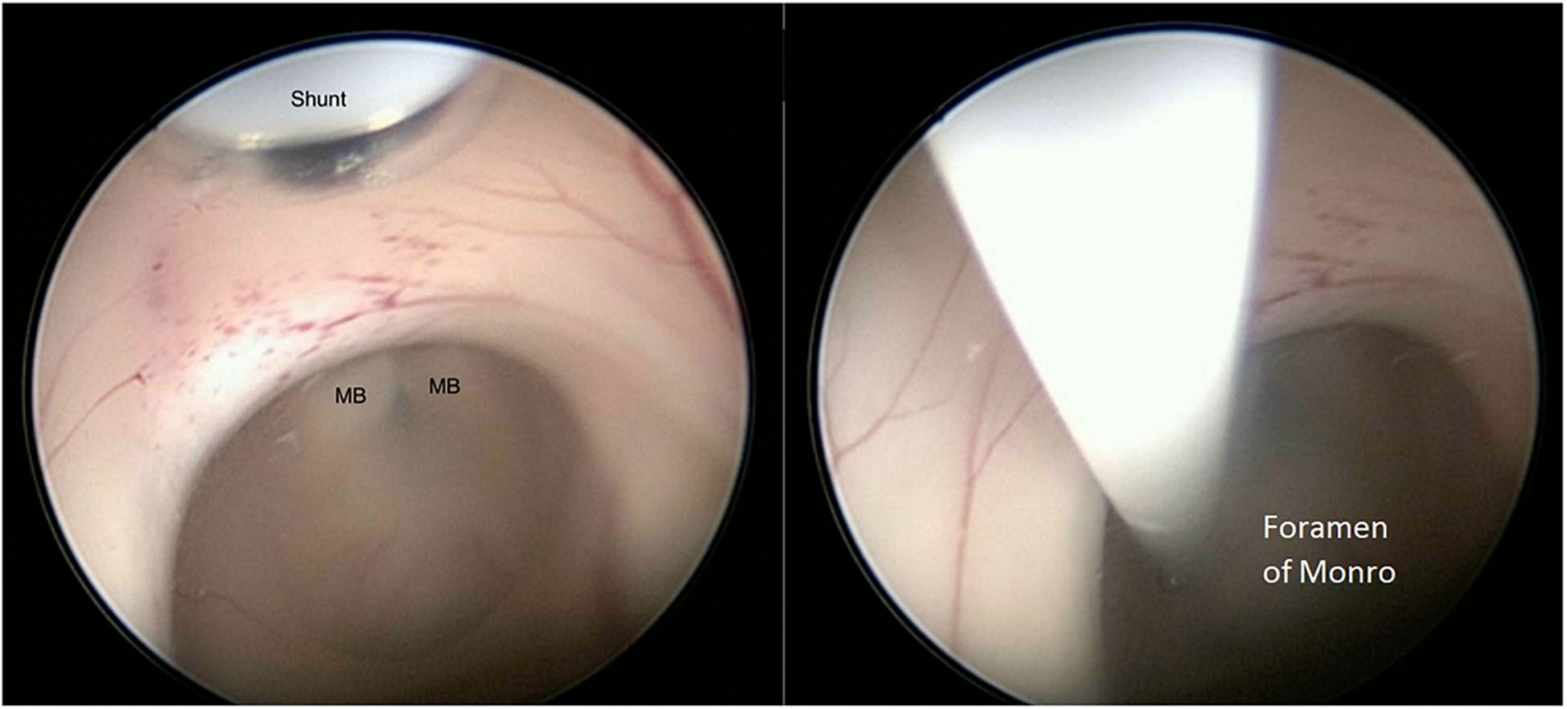
The ventricular catheter is inserted into the right lateral ventricle through the endoscope channel. The catheter tip is positioned inside the III ventricle passing through the foramen of Monro. Abbreviations: MB: Mammillary bodies

For the placement of the ventricular catheter, the head is in a neutral position and not fixed. A C shaped or linear incision is performed over the entry point previously planned with the electromagnetic neuronavigation system. A precoronal burr hole is performed on the mid-pupillary line even if the position could vary based on the ventricular size and morphology and on the anatomic variability of the head which is greater in children then in adults. The dura mater is punched and coagulated using the monopolar. A standard ventricular catheter is positioned into the third ventricle through the foramen of Monro. The neuroravigation using the proper stylet positioned near to the endoscope is useful to maintain the trajectory planned preoperatively (Fig. 1) and the catheter is then inserted into the right lateral ventricle through the endoscope channel or coaxial to the endoscope using the same burr hole, depending on the catheter size. The endoscope could help in the correct positioning of the catheter tip into the III ventricle (Fig. 2). The catheter in then anchored to the pericranium to avoid displacements.

**Fig. 3:**
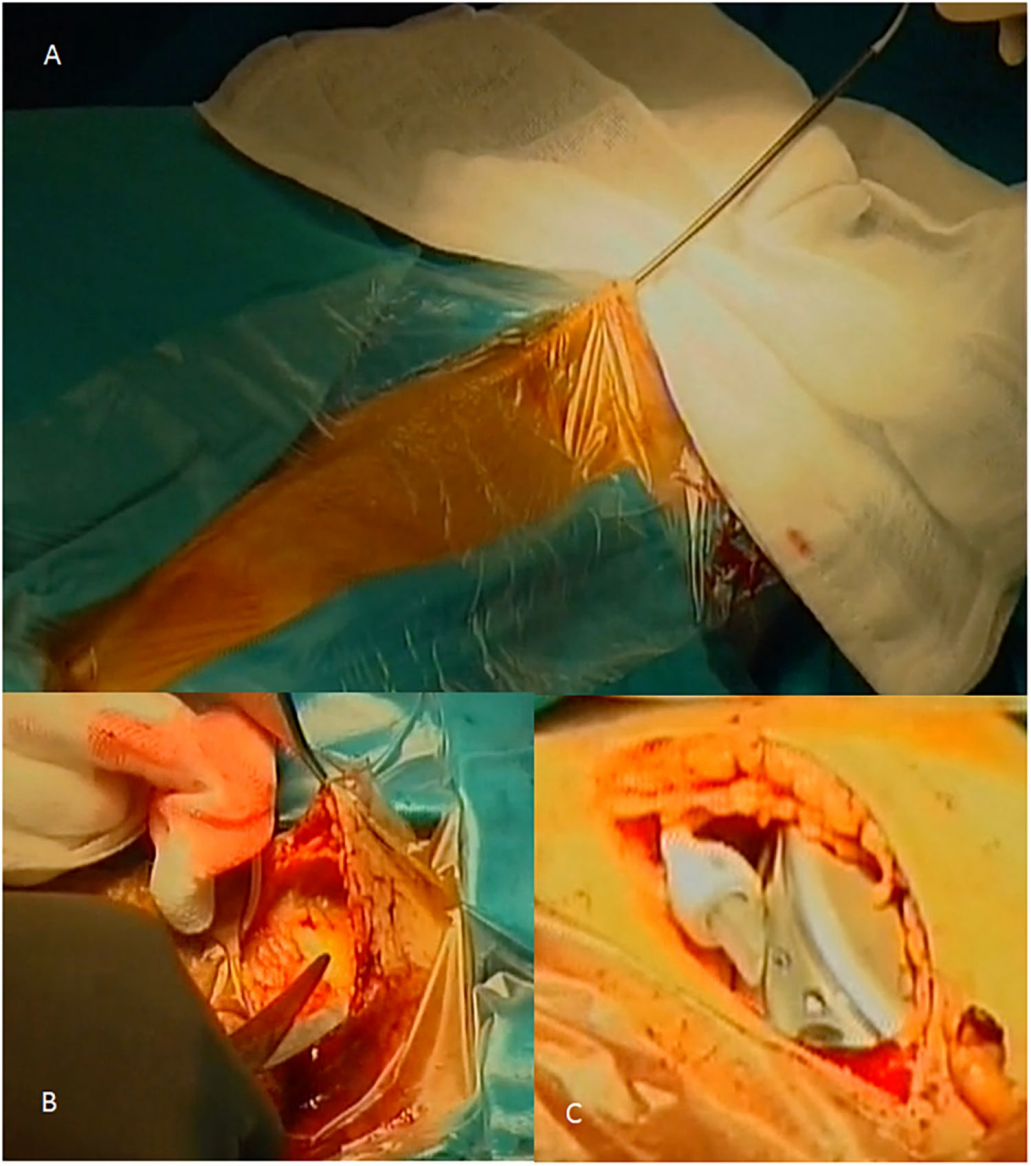
**A** A disposable catheter passer is used for a ‘double-pass’ shunt tunneling from the abdominal incision to the frontal one. **B** A subcutaneous pocket is created using scissors and (**C**) the previously filled pump for drug delivery, connected to the shunt, is positioned inside the pocket

For the tunneling a contralateral rotation of the head is needed to expose the retromastoid portion of the scalp and to facilitate the tunneling procedure. A 7 cm linear incision is performed in the lateral abdominal homolateral wall 2 cm below the umbilical line. A disposable catheter passer (CODMAN®, Integra LifeSciences, Princeton, NJ, USA) is used for a ‘double-pass’ shunt (Medtronic catheter, model 8731sc) tunneling from the abdomen to the head or viceversa. The ventricular catheter is then connected to the distal portion using a straight connector provided with the catheter. The shunt is then connected to a previously filled pump for drug delivery (Synchromed II, Medtronic Inc., Minneapolis, MN-55440, USA), implanted in a subcutaneous pocket (Fig. 3). The drug infusion rate was 0.5 ml/day (250.000 I.U. die).

## Indications

**Fig. 4:**
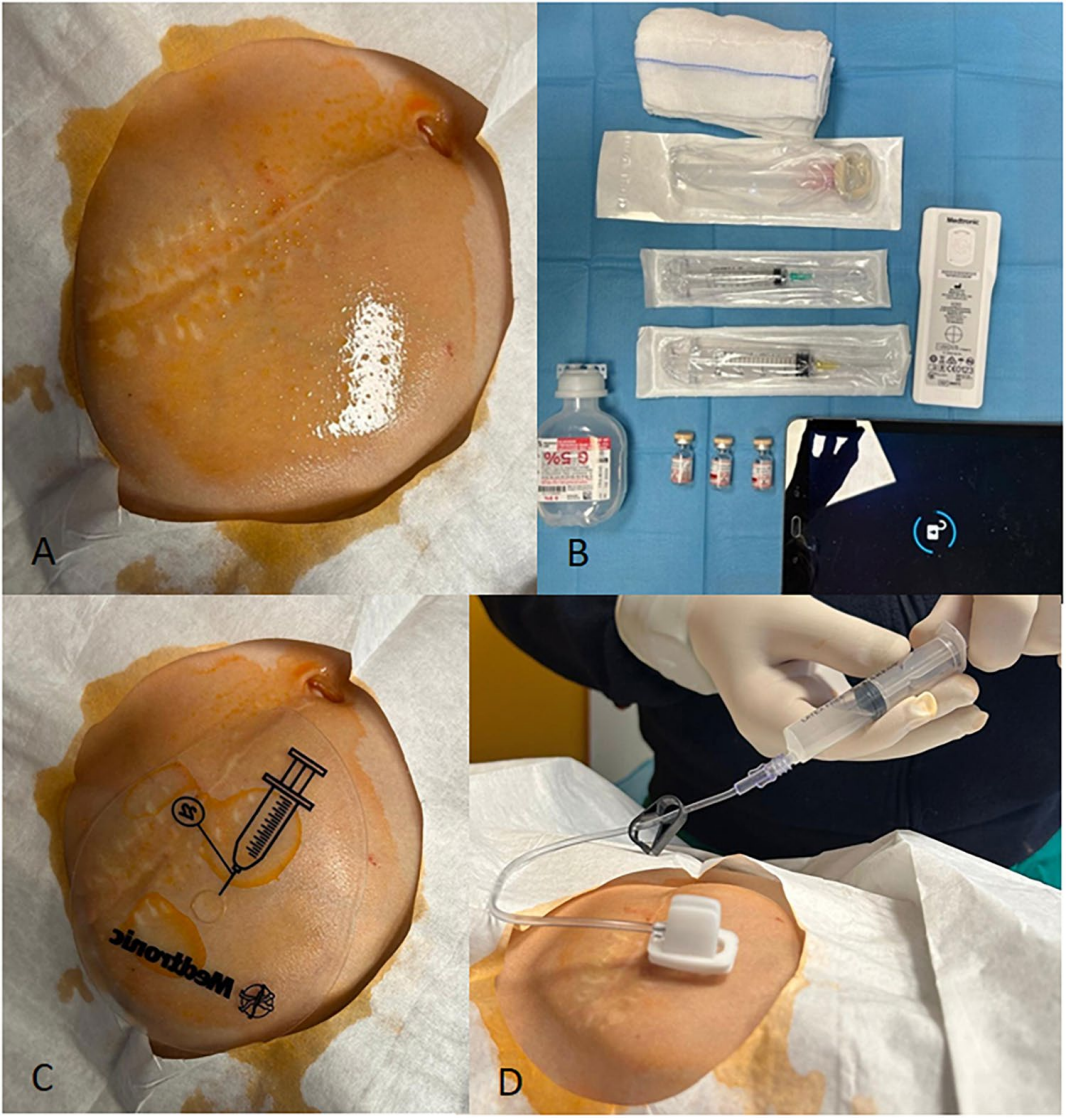
Pump refill: the site is sanitized (**A**), all the instruments and drugs are prepared (**B**), the soft groove in the middle of the pump is identified and punched (**C**), the drug is then injected in the reservoir after emptying it from the residue (**D**)

**Fig. 5:**
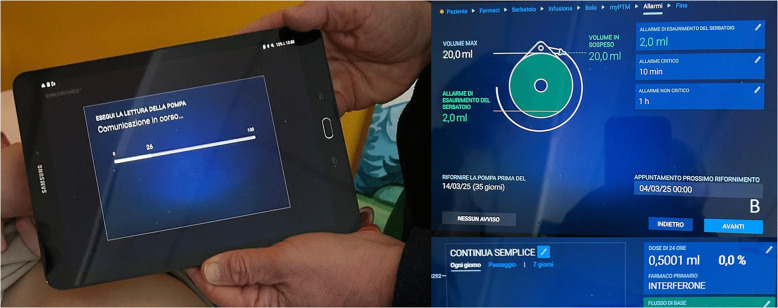
Pump settings: The pump is connected to the software (**A**), the reservoir volume is 20 ml (**B**) and the daily dose of α-IFN is 0,5001 ml (0.0208 ml/h) (**C**)

Since SSPE is a rare neurodegenerative condition, an accurate diagnosis should be made and a multidisciplinary team discussion is necessary for choosing the best treatment. The use of the subcutaneous rechargeable pump permits a continuous drug administration and a consequent maintaining of a constant cerebral drug concentration reducing the toxicity of the medication. The pump is refilled with 9.000.000 I.U. of α-IFN approximately every 21 days (Figs. 4 and 5). In the literature, data on treatment duration are limited, as survival beyond four years from disease onset is rare. However, particularly when initiated in the early stages, lifelong treatment may be advisable to help maintain stabilization of the neurological condition [9].

## Limitations

Contraindication of the placement of a ventricular shunt connected to a rechargeable pump are infections of the CSF or over the entry sites (abdominal and cranial). Slit-like ventricles could make the procedure difficult but they are not a contraindication.

## Specific perioperative considerations

Neuronavigation-guided ventricular catheter placement is useful in all patients and especially for puncturing slit-like ventricles. An accurate preoperative planning is essential for calculating the length of the catheter and the positioning of the tip in the III ventricle. The endoscope could be used as a supplementary tool helping the placement and avoiding bleeding. The catheter tunneling could be done from the head to the belly or vice-versa depending on the surgeon choice. The technique described promotes appropriate drug administration favoring the α-IFN flow to the cerebral convexities avoiding the stagnation in the lateral ventricle.

## Specific information to give to the patient about surgery and potential risks

Whereas the patients are minors, the information is given to parents.

The placement of a catheter connected to a rechargeable pump aims at continuous delivering intraventricular α-IFN. Although the rate of infection is lowered than by repeated puncturing of a reservoir, the possible complications of the procedure include CSF and wound infections and revision surgery up to a removal of the system may be required. Intraventricular bleeding may occur with the possibility of reintervention. In addition, parents should be informed that the pump must be periodically refilled and the battery changed when exhausted.

They are also informed about the risk that their child may not experience improvement of the preoperative symptoms, however, in our experience, deficits generally tend to improve in the postoperative period. It is important that parents are psychologically ready to face their children illness.

Adverse effects of α-IFN therapy are generally non-specific and can include reduced appetite, pyrexia, fatigue, and aseptic (chemical) meningitis. Long-term and repeated administration may heighten the risk of interferon-α–related encephalopathy, as well as neurotoxicity affecting both upper and lower motor neurons. Routine blood tests, including virological screening, are recommended as pre-treatment laboratory investigations. In post-treatment monitoring, assessing organ function indices, particularly liver function indices, is useful [6].

## Ten key points

1. Subacute sclerosing panencephalitis (SSPE) is a rare and severe neurodegenerative disorder. The diagnosis is based on Dickens’criteria although a multidisciplinary team discussion is essential to guarantee a correct management.
2. An intraventricular α-IFN infusion through a subcutaneous rechargeable pump guarantees a constant amount of drug without the need of several punctures to administer it lowering the risk of infection.
3. The right frontal entry point is preferred so the position of the catheter tip would be in the III ventricle passing through the foramen of Monro to maximize the drug effect.
4. The neuronavigation stylet is inserted into the endoscope channel helping to follow the planned trajectory to the right lateral ventricle.
5. The endoscope could help in identifying the foramen of Monro the third ventricle and the fornix in order to avoid complications and check the right positioning of the catheter.
6. The position of the subcutaneous pump should be in the right lateral abdominal wall to avoid bedsores and to facilitate the refill.
7. The tunneling is performed from the abdomen to the head or viceversa passing in the subcutaneous plane and paying attention to pass over the costal plane and clavicle which should be used as a reference.
8. The reload should be done 3–4 days before the exhaustion of the drug reserve. The pump refill occurs every approximately 21 ays with 9.000.000 I.U. of α-IFN.
9. In the refill procedure, with a sterile field, the soft groove in the middle of the pump is identified and punched, the reservoir is emptied from the residual drug and the “new” α-IFN is injected until filled.
10. In our experience this technique results in an improvement of the clinical and neuroradiological outcome reducing drug toxicity and infections.

## Supplementary information

Below is the link to the electronic supplementary material.

ESM 1 (MP4 34.6 MB)

## Data Availability

No datasets were generated or analysed during the current study.
